# Epigenetic (re)programming of gene expression changes of CB1R and FAAH in the medial prefrontal cortex in response to early life and adolescence stress exposure

**DOI:** 10.3389/fncel.2023.1129946

**Published:** 2023-02-22

**Authors:** Arijana Demaili, Anna Portugalov, Michal Dudai, Mouna Maroun, Irit Akirav, Katharina Braun, Jörg Bock

**Affiliations:** ^1^Department of Zoology and Developmental Neurobiology, Institute of Biology, Otto von Guericke University Magdeburg, Magdeburg, Germany; ^2^Department of Psychology, School of Psychological Sciences, University of Haifa, Haifa, Israel; ^3^The Integrated Brain and Behavior Research Center, University of Haifa, Haifa, Israel; ^4^Sagol Department of Neurobiology, Faculty of Natural Sciences, University of Haifa, Haifa, Israel; ^5^Center for Brain and Behavioral Science, Magdeburg, Germany; ^6^Project Group (PG) Epigenetics and Structural Plasticity, Institute of Biology, Otto von Guericke University Magdeburg, Magdeburg, Germany

**Keywords:** endocannabinoid system, DNA-methylation, epigenetics, early life stress, prefrontal cortex

## Abstract

Environmental factors, including stress, that are experienced during early life (ELS) or adolescence are potential risk factors for the development of behavioral and mental disorders later in life. The endocannabinoid system plays a major role in the regulation of stress responses and emotional behavior, thereby acting as a mediator of stress vulnerability and resilience. Among the critical factors, which determine the magnitude and direction of long-term consequences of stress exposure is age, i.e., the maturity of brain circuits during stress exposure. Thus, the present study addressed the hypotheses that ELS and adolescent stress differentially affect the expression of regulatory elements of the endocannabinoid system, cannabinoid receptor 1 (CB1R) and fatty acid amide hydrolase (FAAH) in the medial prefrontal cortex (mPFC) of adult female rats. We also tested the hypothesis that the proposed gene expression changes are epigenetically modulated *via* altered DNA-methylation. The specific aims were to investigate if (i) ELS and adolescent stress as single stressors induce changes in CB1R and FAAH expression (ii) ELS exposure influences the effect of adolescent stress on CB1R and FAAH expression, and (iii) if the proposed gene expression changes are paralleled by changes of DNA methylation. The following experimental groups were investigated: (1) non-stressed controls (CON), (2) ELS exposure (ELS), (3) adolescent stress exposure (forced swimming; FS), (4) ELS + FS exposure. We found an up-regulation of CB1R expression in both single-stressor groups and a reduction back to control levels in the ELS + FS group. An up-regulation of FAAH expression was found only in the FS group. The data indicate that ELS, i.e., stress during a very immature stage of brain development, exerts a buffering programming effect on gene expression changes induced by adolescent stress. The detected gene expression changes were accompanied by altered DNA methylation patterns in the promoter region of these genes, specifically, a negative correlation of mean CB1R DNA methylation with gene expression was found. Our results also indicate that ELS induces a long-term “(re)programming” effect, characterized by CpG-site specific changes within the promoter regions of the two genes that influence gene expression changes in response to FS at adolescence.

## Introduction

The perinatal period is a critical phase for brain development, characterized by increased susceptibility to environmental factors, including adverse experiences such as early life stress (ELS) (McEwen, 2006; Bock et al., 2014; Babenko et al., 2015). ELS represents a critical environmental factor underlying the etiology of mental disorders (Green et al., 2010; St Clair et al., 2015; Liu, 2017). On the other hand, there is an increasing evidence that ELS can also induce adaptive mechanisms promoting stress resilience later in life (Brockhurst et al., 2015; Gröger et al., 2016a). Studies in humans, non-human primates and rodents consistently show that specifically the development of limbic and prefrontal-cortical brain circuits is particularly sensitive toward ELS exposure (Bock and Braun, 2011; Malter Cohen et al., 2013; Suzuki et al., 2014; Johnson et al., 2018; Smith and Pollak, 2020). Maternal neglect or repeated maternal separation in rodents are widely applied and established experimental animal models to evaluate the effect of ELS on the molecular, brain structural and behavioral level (Wang et al., 2012; Gröger et al., 2016b; Walker et al., 2017; Köhler et al., 2019). However, it is important to keep in mind that in “real” life exposure to stressors are not single events but usually occur consecutively during different life spans. This raises the critical question if and in which way individual’s responses to consecutive stressors might interact and thereby either lead to an increased (vulnerability) or decreased (resilience) stress responsiveness. The “two-(or multiple) hit” concept (Lupien et al., 2009; Gruber et al., 2015; Lesse et al., 2017; Santarelli et al., 2017; Jaric et al., 2019) claims that early adverse experiences such as ELS may have an “programming” effect on stress responses and sensitivity to adverse experiences later in life (Nederhof and Schmidt, 2012). However, the mechanisms of these complex interactions and how they may promote vulnerable or resilient adaptations are far from understood in detail. There is increasing evidence that epigenetic mechanisms may act as mediators between genetically programmed developmental processes and potentially (re-)programming early environmental influences (Bock et al., 2014; Köhler et al., 2019; Kocamaz et al., 2022; Rahman and McGowan, 2022). Thus, they are discussed to play a crucial role in translating early adverse experiences into long-lasting gene expression changes and thereby can modulate stress-related responses (Bock et al., 2014; Provençal and Binder, 2015; Radtke et al., 2015; Alyamani and Murgatroyd, 2018; Li et al., 2020).

Candidates which may be essentially involved in the long-term consequences of ELS include the endocannabinoid system and their receptors (Marco et al., 2014; Lutz, 2020; Goldstein Ferber et al., 2021). Endocannabinoids (eCBs) are neurotransmitters of lipid origin that act as endogenous ligands for cannabinoid receptors (CBRs), they are not stored in vesicles, but rather released “on demand.” Two main eCBs have been described so far, N-arachidonoylethanolamine (anandamide, AEA) (Devane et al., 1992) and 2-arachidonoylglycerol (2-AG) (Mechoulam et al., 1995; Sugiura et al., 1995). The metabolic control of eCB signaling is generated in part by degradation enzymes, fatty acid amide hydrolase (FAAH), involved in the degradation of AEA, and by monoacylglycerol lipase (MAGL), for 2-AG (Egertová et al., 1998; Romero et al., 2002; Maccarrone, 2017). The endocannabinoid receptor 1 (CB1R) is a G protein-coupled receptor, located presynaptically on GABAergic and glutamatergic neurons and is thus involved in the retrograde signaling of neurotransmission. CB1R is highly expressed in brain areas, which are involved in the control of emotional behavior and in memory-related plasticity such as the prefrontal cortex and amygdala and the hippocampal formation (Mechoulam and Parker, 2013; Pucci et al., 2021).

A widely established and applied model of ELS, to mimic early life adversities including neglect, abuse and psychosocial stress in humans, is maternal neglect in rats and mice during early postnatal development (Chen and Baram, 2016; Short and Baram, 2019). Applying this model it was found that aberrant patterns of maternal care influence the maturation and refinement of functional synaptic and neuronal circuits resulting in impairments of spatial and recognition memory as well as in symptoms of mental disorders. For the present study we applied a slightly modified maternal neglect paradigm that previously has been shown to induce impairments in social preference and social recognition as well as enhanced depression-like and anxiety-like behavior in adult rats (Alteba et al., 2016, 2020, 2021; Portugalov et al., 2022). These studies also showed that enhancing eCB signaling by pharmacological treatment during late adolescence restored ELS-induced depressive-like behavior and alterations in eCB signaling, which were associated with the behavioral phenotype. Interestingly, restorage of stress-induced changes of FAAH after pharmacological treatment could only be observed in females but not in males. The role of the eCB system in anxiety and depression was also demonstrated in other studies using different stress paradigms, however, the exact molecular mechanism and specifically the underlying epigenetic modifications are still not elucidated in detail. Accordingly, one aim of this study was to test the hypothesis that ELS as well as adolescent stress (forced swimming; FS) as single stressors in female rats induce changes in gene expression of two main regulatory components of the eCB system, CB1R and FAAH, and that these changes are epigenetically mediated *via* altered DNA-methylation at the respective gene promoters. Moreover, we addressed the hypothesis that ELS exerts a (re-)programming effect on gene expression changes induced by FS *via* CpG-site specific alterations of DNA-methylation. To test this, we analyzed the effects of combined exposure to the two stressors on the expression of the CB1R and FAAH genes, the CpG-site specific DNA-methylation in the promoter regions of these genes and a comparison with the effects of the single stress exposures.

## Materials and methods

### Animals

Sprague–Dawley (SD) rat dams (Envigo, Israel) and pups were housed in the animal facility of the School of Psychological Sciences, University of Haifa. Animals were housed in a polypropylene cage (59 × 28 × 20 cm), on a 12:12 light/dark cycle with food and water provided ab libitum, while room temperature was 22 ± 2. Pups were weaned on PND21 (day of birth was defined as PND0), and randomly assigned to the same sex groups (4–5 animals per cage). For this study we focused on the female groups, males were used for parallel experiments. From each litter, no more than one female was randomly assigned to each experimental condition (all female pups were used in the different treatment groups). The experiments were approved by the University of Haifa Ethics and Animal Care Committee and appropriate measures were taken to minimize pain and discomfort (approval numbers: 741/20, 765/21).

### Stress paradigms

#### Early life stress (ELS)

As ELS an established paradigm was applied that induced maternal neglect and fragmented maternal care by inappropriate housing conditions (Avishai-Eliner et al., 2001) with modifications (Alteba et al., 2016, 2020, 2021; Portugalov et al., 2022). The dam and her pups were housed in a cage with limited “Sunny Chips” bedding material (1.2 cm layer) from PND7 to PND14. The no ELS dam and her pups were housed in a cage with abundant (7–9 cm layer) bedding material.

#### Adolescence stress (forced swimming, FS)

Stress in adolescence was induced by exposing rats to swim stress (forced swimming, FS) on two consecutive days (PND 28-29). Experimental animals were forced to swim in an acrylic cylindrical container (62 cm diameter, 40 cm height), filled with water (temperature 24°C). On the first day, the FS lasted for 15 min, after which the rat was returned to the home cage. On the following day the rats were subjected to 5 min FS.

### Experimental groups

#### Female rats were assigned to the following four experimental groups

Control (CON): were not exposed to any stressor and were housed in cages with adequate bedding material (7 to 9 cm layer). The pups were housed with their mother under non-stressful conditions until weaning.

Early life stress (ELS): were exposed to maternal neglect as described above from PND 7-14.

Forced swimming (FS): were reared as controls but were exposed to forced swim stress in early adolescence (PND 28-29).

ELS + FS: were exposed to both stressors, ELS and FS.

Rats from the same experimental groups were group housed, therefore rats from different experimental groups were kept in separate cages.

### Behavioral tests

All rats were exposed to the same battery of behavioral tests in adulthood. Testing occurred under dim lighting (15–20 lx) and took place between 13:00 and 16:00 h. Rats were randomly assigned to the respective experimental groups (CON, *n* = 16; ELS, *n* = 12; FS, *n* = 12; ELS + FS, *n* = 14).

### Activity and anxiety-like behavior in an open field test (OF)

The apparatus consists of a square black open field (50 × 50 × 50 cm). The floor is divided by 1 cm wide white lines into 25 squares measuring 10 × 10 cm each. The open field arena was thoroughly cleaned between each trial. The movements of the rat were recorded and analyzed for 15 min using a video tracking system (Ethovision × T 14.0, Noldus Information Technology) to measure locomotion (measured as distance moved in cm). Time spent in the center (s) in the first 5 min was used as a measure of anxiety-like behavior (Bauminger et al., 2022).

### Social preference and social recognition

This task aims to assess sociability and short-term social memory. The “partner” rat was confined to a separate section of the open field (50 cm× 50 cm× 50 cm) by a transparent perforated Plexiglas panel (corrals) (Portugalov et al., 2022). For the *preference* phase, the experiment rat was given 5 min exploration with a novel juvenile and a novel object. For the *recognition* phase, after 30 min in a holding cage, the rat was given 5 min exploration with the familiar juvenile and a novel juvenile, both confined to the corrals. The trials were videotaped (Dericam, Indoor Pan/tilt IP camera M801W, USA). An exploration index was calculated; for social preference: time exploring the novel juvenile/total exploration time (object + juvenile rat). For social recognition: time exploring the novel juvenile/total exploration time (familiar + novel juvenile).

### Activity and anxiety-like behavior in the elevated plus maze (EPM)

The apparatus consists of black plus-shaped maze (110 × 110 cm, 70 cm above the floor; two opposing open arms/closed arms). The rat was placed in the center of the maze and allowed to explore the maze freely for 5 min. The movements of the rat were recorded and analyzed using a video tracking system (Ethovision × T 14.0, Noldus Information Technology) to measure locomotion (measured as distance moved in cm). Freezing (s) and time spent in the closed arms (s) were used as a measure of anxiety-like behavior.

### Saccharine preference test

Water bottles were removed before the dark part of the cycle and replaced with two bottles, one filled with water and the other with saccharine (0.3 mg/l) dissolved in water. Saccharine consumption was measured during the 12 dark hours of the cycle and then normalized according to every rat’s specific weight. The saccharine preference *ratio* was calculated as the saccharine consumption divided by the total consumption (saccharine consumption + water consumption). The measurements of saccharine preference were taken 3 times with 24 h interval between them. An average of all the measurement were calculated for each rat.

### Behavioral profiling

In order to classify the stress-exposed animals as “affected” or “unaffected” behavioral profiling was conducted based on the cut-off behavioral criteria analysis as described previously (Ardi et al., 2016; Albrecht et al., 2021; Sarkar et al., 2022). All the behaviors described above were used as key parameters. The performance of the control group was analyzed as the behavior of an unaffected “normal population,” and average and standard deviation were calculated to build the upper and lower cut-offs for each parameter. Different behavioral profiles were then built, classifying stress-exposed animals as “affected” if they fell under or above the lower and upper cut-off values in more than 3 tests.

### Tissue preparation

Animals of the respective experimental groups were sacrificed by decapitation on PND110, between 6 and 9 a.m. Brains were immediately collected, frozen in liquid nitrogen and stored at −80. Prior to gene expression analysis, right and left medial prefrontal cortex (mPFC) tissue was dissected. Tissue for gene expression and DNA methylation analyses was derived from the same animals (right hemisphere mPFC used for RNA extraction, and left mPFC for DNA extraction).

Total RNA was extracted using the innuPREP RNA Mini Kit 2.0 (Analytik Jena, Berlin, Germany) according to the manufacturer’s instructions. The genomic DNA contaminants were removed using the innuPREP DNase I Digest Kit (Analytik Jena, Berlin, Germany).

For analysis of gene expression and DNA-methylation rats were randomly assigned to the respective experimental groups (CON, *n* = 9; ELS, *n* = 9; FS, *n* = 8; ELS + FS, *n* = 9).

### Gene expression analysis

One-step quantitative real-time PCR was performed using the QuantiNova Multiplex RT-PCR kit (Qiagen GmbH, Hilden, Germany) and Taqman gene expression assays (Life Technologies, Carlsbad, CA, USA). Commercially available rat Taqman probes were used for cannabinoid receptor 1 (CB1R, *cnr1* gene) and fatty acid amide hydrolase (FAAH) as target genes (Rn00562880_m1 for *cnr1* and Rn00577086_m1 for *faah*) and hypoxanthine guanine phosphoribosyl transferase (HPRT) as reference gene (Rn01527840_m1). The relative gene expression was calculated using the delta-delta Ct method (Livak and Schmittgen, 2001) after normalization using the reference gene. All samples were run in 2 independent experimental replicates, and in triplicates within one gene assay.

### DNA-methylation analysis

Deoxyribonucleic acid methylation levels were determined using the bisulfite-converted DNA and pyrosequencing method (Kocamaz et al., 2022). In short, genomic DNA was extracted from left mPFC using DNeasy Blood and Tissue Kit (Qiagen GmbH, Hilden, Germany) according to the manufacturer’s instructions. EpiTect Bisulfite Kit (Qiagen GmbH, Hilden, Germany) and QIAcube (Qiagen GmbH, Hilden, Germany) were used for DNA bisulfite conversion and cleanup, respectively. Analyzed DNA sequences of the CB1R and FAAH genes were identified by using the National Center for Biotechnology Information (NCBI) databank. Primers covering 11 CpG sites in the promoter region of the CB1R gene were individually designed using PyroMark Assay Design Software (Qiagen GmbH, Hilden, Germany). For the FAAH gene, 2 primers covering 9 CpG sites within the promoter region were used, which are commercially available as PyroMark CpG assays (Qiagen). The specificity of custom-made and predesigned primers was confirmed by capillary electrophoresis. The genomic location of each CpG site was determined according to their distance from the start codon (Tables 1–4). Pyrosequencing was performed with Pyromark Q96 ID (Qiagen GmbH, Hilden, Germany), using Pyromark Q96 Gold reagents (Qiagen GmbH, Hilden, Germany). For verification of methylation results, CpGenome rat methylated and unmethylated genomic DNA standards (Merck KGaA, Darmstadt, Germany) were used. DNA methylation levels were automatically analyzed using PyroQ CpG software (Qiagen GmbH, Hilden, Germany).

**TABLE 1:** Designed CB1R primers used for DNA methylation.

	Distance from start codon	Primers (5′–3′)	Sequence (Bold text–CpG-sites analyzed)
P1	−18,770 to −18,759	Forward primer: GTGGTAGAGAGTGAGGATGATATA Reverse primer (biotinylated): CTAACCCCAAAACTAAAAAAACCAACC Sequencing primer: AGAGTGAGGATGATATAG	GA**CG**C**CG**AG**CG**A
P2	−18,088 to −18,051	Forward primer: GGGGGTAAATTTTTTTGTAGTAGAGA Reverse primer (biotinylated): ACCAAATCTTACCCTTCTCAT Sequencing primer: TTTGTAGTAGAGAGTTAGTT	GG**CG**ACAGGTGC**CG**AGGGAGCTTCTGGCC**CG**TGGAC**CG**
P3	−17,953 to −17,936	Forward primer: TGAGAAGGGTAAGATTTGGTATAGTG Reverse primer (biotinylated): CCTCCAAAAATAACTTAAACCACAAAT Sequencing primer: GGTTAGTGGTTTTTAGGT	**CGCG**GAGTT**CG**TGTTC**CG**

**TABLE 2:** Location of CB1R individual CpG sites analyzed in DNA methylation.

Individual CpG-site	Distance from start codon
1–P1	−18,768
2–P1	−18,765
3–P1	−18,761
4–P2	−18,086
5–P2	−18,076
6–P2	−18,059
7–P2	−18,052
8–P3	−17,953
9–P3	−17,951
10–P3	−17,944
11–P3	−17,937

**TABLE 3:** FAAH gene primers analyzed in DNA methylation.

	Distance from start codon	Primers (5′-3′)	Sequence (Bold text–CpG-sites analyzed)
P1	−289 to −253	Rn_Faah_01_PM PyroMark CpG assay, GeneGlobe ID: PM00559699	C**CG**GC**CG**CCC**CG**GTGTTTTTGAGGGTGCCCA**CGCG**GA
P2	+ 344 to +385	Rn_Faah_02_PM PyroMark CpG assay, GeneGlobe ID: PM00559706	**CG**GTCCTCTTTCTG**CG**CCAGGTGCAG**CG**CAAGGGGAAA**CG**GA

**TABLE 4:** Position of individual CpG-sites within the promoter region of FAAH gene used for DNA methylation analysis.

Individual CpG-site	Distance from start codon
1–P1	−288
2–P1	−284
3–P1	−279
4–P1	−258
5–P1	−256
6–P2	+ 344
7–P2	+ 358
8–P2	+ 370
9–P2	+ 382

### Statistics

Differences between the experimental groups regarding gene expression, mean DNA methylation and CpG site specific methylation were analyzed using a 2-way analysis of variance (ANOVA), with ELS and FS as main factors. For more detailed differences between the individual groups, Student Newman Keuls (SNK) *post hoc* was used. In addition, Pearson correlation analysis was used to calculate the correlation between CB1R and FAAH gene expression results, and to calculate the correlation between gene expression and DNA methylation results. Individual behavioral profiling was applied as described above and the distribution of affected vs. unaffected populations was calculated by using Pearson’s chi-squared test. All statistical calculations were performed using GraphPad Prism 8 (San Diego, CA, USA), with the exception of behavioral profiling, which was analyzed using SPSS 27 (IBM, Chicago, IL, USA). All values were presented as mean value ± SD. P < 0.05 was considered statistically significant.

## Results

### Behavioral tests

#### Open field test (OF) and elevated plus maze (EPM)

**FIGURE 1:**
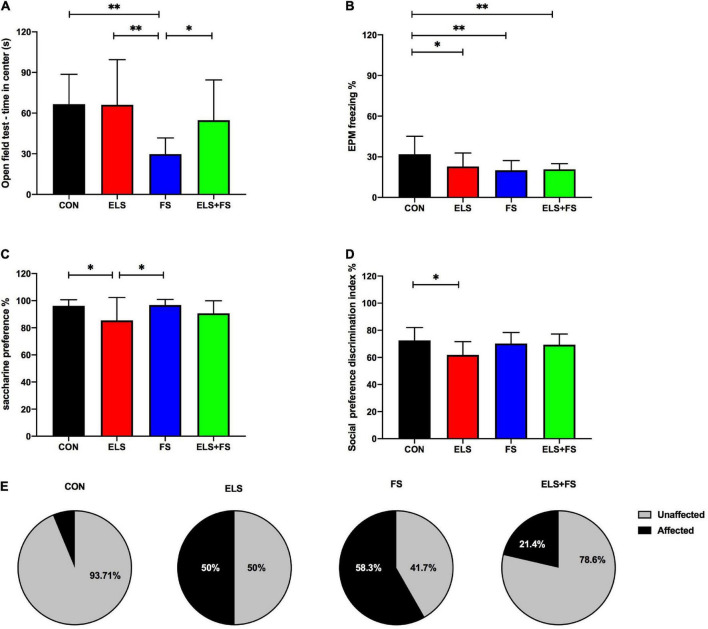
The effects of ELS and FS on anxiety-like behavior, depression-like behavior and social preference in the adult female rats. The effects of different behavioral parameters are calculated using 2-way ANOVA (see text) and significant results of SNK *post-hoc* test are indicated as *p ≤ 0.05 and ^**^p ≤ 0.01. **(A)** OF center time; **(B)** EPM freezing percentage; **(C)** saccharine preference; **(D)** social preference discrimination index; **(E)** percentage distribution of affected and non-affected individuals as a result of behavioral profiling. All bars depict mean ± SD. CON, control (*n* = 16); ELS, early life stress (*n* = 12); FS, forced swimming (*n* = 12); ELS + FS, early life stress + forced swimming (*n* = 14).

For the time spent in the center of the OF, 2-way ANOVA revealed a significant main effect for FS [*F*(1,50) = 11.91, *p* = 0.0011], and a trend toward significance for ELS [*F*(1,50) = 3.118, *p* = 0.0835] as well as for interaction [*F*(1,50) = 3.322, *p* = 0.0743]. The SNK *post-hoc* test for individual group comparisons revealed FS animals spent significantly less time in center of OF when compared to CON (*p* ≤ 0.01), ELS (*p* ≤ 0.01) and ELS + FS (*p* ≤ 0.05) groups (Figure 1A). No significant differences were found between other groups. For the overall distance covered in the open field test, 2-way ANOVA analysis revealed a strong trend toward significance for ELS [*F*(1,50) = 3.893, *p* = 0.054], but no effects for FS [*F*(1,50) = 0.241, *p* = 0.6257] and interaction [*F*(1,50) = 0.3193, *p* = 0.5745].

For the EPM, 2-way ANOVA analysis revealed a significant main effect for FS on freezing time [*F*(1,50) = 7.234, *p* = 0.0097], a trend toward significance for interaction [*F*(1,50) = 3.564, *p* = 0.0648] but no clear main effect for ELS [*F*(1,50) = 2.533, *p* = 0.1178]. The SNK *post-hoc* test revealed that compared to CON, all other experimental groups showed less freezing time in the EPM (ELS, *p* ≤ 0.05; FS, *p* ≤ 0.01; ELS + FS, *p* ≤ 0.01) (Figure 1B).

For the time spent in closed arms, 2-way ANOVA analysis revealed a main significant effect for ELS [*F*(1,50) = 4.452, *p* = 0.0399], but no effect for FS [*F*(1,50) = 0.374, *p* = 0.5435] and interaction [*F*(1,50) = 0.038, *p* = 0.8456]. The SNK *post-hoc* test revealed no significant difference between the groups. For the running distance covered in the elevated plus maze, 2-way ANOVA analysis revealed no significant effects for ELS [*F*(1,50) = 0.079, *p* = 0.7787], FS [*F*(1,50) = 1.176, *p* = 0.2834] and no interaction [*F*(1,50) = 1.404, *p* = 0.2417].

#### Saccharine preference test and social preference test

For saccharine preference test, 2-way ANOVA analysis showed a significant main effect for ELS [*F*(1,50) = 10.07, *p* = 0.0026], but no main effect for FS [*F*(1,50) = 1.212, *p* = 0.2763] and no interaction [*F*(1,50) = 0.797, *p* = 0.3761]. The SNK *post-hoc* test revealed that ELS group has a lower saccharine preference compared to CON (*p* ≤ 0.05) and FS (*p* ≤ 0.05) (Figure 1C). No significant differences were found between other groups.

For the discrimination index in the social preference test, 2-way ANOVA analysis revealed a significant main effect for ELS [*F*(1,50) = 5.747, *p* = 0.0203], a strong trend toward significance for interaction [*F*(1,50) = 3.941, *p* = 0.0526] and no effect for FS [*F*(1,50) = 1.145, *p* = 0.2898] The SNK *post-hoc* test revealed that the ELS group has a lower discrimination index compared to CON group (*p* ≤ 0.05) (Figure 1D). For the discrimination index in the social recognition test, 2-way ANOVA analysis showed no significant effects for ELS [*F*(1,50) = 0.497, *p* = 0.484], FS [*F*(1,50) = 0.573, *p* = 0.452] and no interaction [*F*(1,50) = 0.303, *p* = 0.584].

#### Different behavioral profiles after ELS and FS exposure

In order to classify the stress-exposed animals as stress-affected or stress-unaffected, an individual behavioral profiling, based on the behavioral parameters described above, was conducted. Chi-Square-test revealed a clear significant difference (Chi^2^ = 11.207, *p* = 0.010) between the groups. While 50% in the ELS and 58.3% in the FS group were affected by stress, only 21.4% were affected in the ELS + FS group and only 1 animal in the CON group (6.23%) could be classified as affected (Figure 1E).

### Relative gene expression

#### Expression of cannabinoid receptor 1 (CB1R) is increased after ELS and FS

Two-way ANOVA analysis revealed no significant main effects for ELS [*F*(1,31) = 0.5555, *p* = 0.4617] or FS [*F*(1,31) = 0.2117, *p* = 0.6487], but a highly significant interaction between these two stressors was found [*F*(1,31) = 18.48, *p* = 0.0002].

**FIGURE 2:**
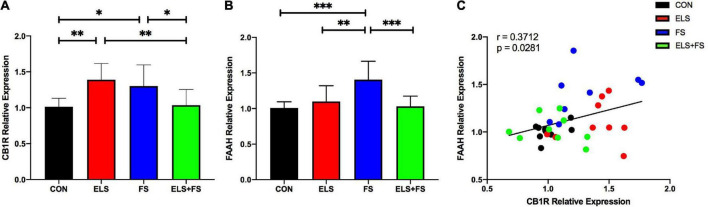
ELS and FS effects on cannabinoid receptor 1 (CB1R) and fatty acid amide hydrolase (FAAH) relative expression in the medial prefrontal cortex (mPFC) of adult female rats. Relative gene expression data are calculated and shown as 2^– DDCt^ values ± SD [CB1R results **(A)**; FAAH results **(B)**]. Significant results of SNK *post-hoc* test are indicated as *p ≤ 0.05, ^**^p ≤ 0.01 and ^***^p < 0.001. Pearson correlation analysis revealed a positive correlation between CB1R and FAAH expression, overal all groups **(C)**. CON, control (*n* = 9); ELS, early life stress (*n* = 9); FS, forced swimming (*n* = 8); ELS + FS, early life stress + forced swimming, (*n* = 9).

The SNK *post-hoc* test for individual group comparisons revealed significantly increased gene expression levels in ELS and FS animals when compared to the CON (ELS: *p* ≤ 0.01; FS: *p* ≤ 0.05) and ELS + FS (ELS: *p* ≤ 0.01; FS: *p* ≤ 0.05) groups (Figure 2A). No significant differences were found between the CON and ELS + FS groups and between the ELS and the FS groups.

#### Expression of fatty acid amide hydrolase (FAAH) is increased after FS

Two-way ANOVA analysis showed a significant main effect for ELS [*F*(1,31) = 5.042, *p* = 0.032], a significant main effect for FS [*F*(1,31) = 6.759, *p* = 0.0142] and a highly significant interaction between these two stressors [*F*(1,31) = 13.69, *p* = 0.0008].

The *post-hoc* tests revealed significantly increased gene expression levels in the FS animals when compared to the other experimental groups, CON (*p* ≤ 0.001), ELS (*p* ≤ 0.01) and ELS + FS (*p* ≤ 0.001) (Figure 2B). No significant differences were found between the CON and the ELS groups, between the CON and the ELS + FS groups and between the ELS and the ELS + FS groups.

#### Correlation of CB1R and FAAH relative gene expression

A Pearson correlation analysis over all the experimental groups revealed a positive correlation between CB1R and FAAH gene expression (*r* = 0.371; *p* = 0.028, Figure 2C), suggesting that increased CB1 expression is associated with increased FAAH expression. Correlation analysis for the individual experimental groups did not reveal significant correlations between expression of the two genes, however, a trend for a positive correlation was found in the CON (correlation *r* = 0.4479, *p* = 0.2267) and the FS group (*r* = 0.4511, *p* = 0.2619). No trend for any correlation was found in the ELS (correlation *r* = 0.1389, *p* = 0.7216) and ELS + FS group (correlation *r* = −0.2033, *p* = 0.5998).

### Stress-induced changes in DNA methylation in the mPFC

#### Cannabinoid receptor 1 (CB1R)

##### ELS and FS decrease mean DNA methylation within the CB1R gene promoter

For DNA methylation analysis, mean methylation levels of 11 CpG sites within the promoter region of the CB1R gene were analyzed. Two-way ANOVA revealed no significant main effects for ELS [*F*(1,31) = 3.448, *p* = 0.0729] or FS [*F*(1,31) = 0.1466, *p* = 0.7044], but a highly significant interaction between the two stressors was found [*F*(1,31) = 25.01, *p* < 0.0001].

**FIGURE 3:**
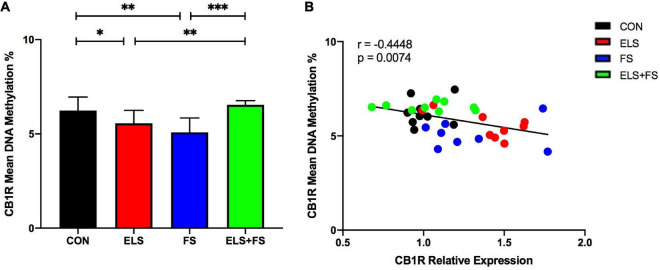
ELS and FS reduce mean DNA methylation levels of the analyzed 11 CpG sites within the promoter region of the CB1R gene. DNA methylation levels are depicted as mean ± SD **(A)**. Significant results of SNK *post-hoc* test are indicated as *p ≤ 0.05, ^**^p ≤ 0.01, and ^***^p ≤ 0.001. Mean DNA-methylation was negatively correlated with CB1R expression in the mPFC of the analyzed experimental groups, ^**^p ≤ 0.01 **(B)**. CON, control (*n* = 9); ELS, early life stress (*n* = 9); FS, forced swimming (*n* = 8); ELS + FS, early life stress + forced swimming (*n* = 9).

The SNK *post-hoc* test displayed a significant decrease in mean DNA methylation levels in the ELS (*p* ≤ 0.05), and FS (*p* ≤ 0.01) groups, when compared to CON group. Also, decreased mean methylation levels were detected in the ELS (*p* ≤ 0.01) and FS (*p* ≤ 0.001) groups when compared to the ELS + FS group (Figure 3A). No significant differences were found between the CON and the ELS + FS groups and between the ELS and the FS groups.

##### Correlation of mean DNA methylation with CB1R gene expression

Pearson correlation analysis over all the experimental groups revealed a negative correlation between mean DNA-methylation and CB1R gene expression (*r* = −0.4448; *p* = 0.0074, Figure 3B), suggesting that increased CB1 expression is associated with decreased DNA-methylation.

##### ELS and FS induce CpG-site specific alterations in DNA methylation within the CB1R gene promoter

Two-way ANOVA analysis for each of the analyzed 11 individual CpG sites within the promoter region of the CB1R gene revealed several significant differences in the methylation levels within the two main factors and a number of significant interactions between the factors (see Table 5 for a summary).

**TABLE 5:** Two-way ANOVA analysis of 11 individual CpG-sites within the promoter region of CB1R gene.

Individual CpG site	Main effect		Interaction
	ELS	FS	ELS + FS
1	No [*F*(1,31) = 0.399, *p* = 0.5325]	No [*F*(1,31) = 0.704, *p* = 0.4079]	**Yes** [*F*(1,31) = 7.002, *p* = 0.0127]
2	No [*F*(1,31) = 1.172, *p* = 0.2874]	No [*F*(1,31) = 0.853, *p* = 0.3628]	**Yes** [*F*(1,31) = 11.690, *p* = 0.0018]
3	No [*F*(1,31) = 0.793, *p* = 0.3801]	No [*F*(1,31) = 0.208, *p* = 0.6517]	**Yes** [*F*(1,31) = 9.688, *p* = 0.004]
4	**Yes** [*F*(1,31) = 11.280, *p* = 0.0021]	No [*F*(1,31) = 2.828, *p* = 0.1027]	**Yes** [*F*(1,31) = 8.492, *p* = 0.0066]
5	No [*F*(1,31) = 0.845, *p* = 0.365]	**Yes** [*F*(1,31) = 5.025, *p* = 0.0323]	No [*F*(1,31) = 0.031, *p* = 0.8617]
6	**Yes** [*F*(1,31) = 9.799, *p* = 0.0038]	No [*F*(1,31) = 3.172, *p* = 0.0847]	**Yes** [*F*(1,31) = 8.093, *p* = 0.0078]
7	**Yes** [*F*(1,31) = 7.817, *p* = 0.0088]	No [*F*(1,31) = 0.446, *p* = 0.5094]	**Yes** [*F*(1,31) = 6.287, *p* = 0.0176]
8	No [*F*(1,31) = 1.062, *p* = 0.3106]	No [*F*(1,31) = 0.070, *p* = 0.793]	No [*F*(1,31) = 0.0133, *p* = 0.909]
9	No [*F*(1,31) = 0.363, *p* = 0.5511]	No [*F*(1,31) = 0.100, *p* = 0.7543]	No [*F*(1,31) = 0.363, *p* = 0.5511]
10	**Yes** [*F*(1,31) = 10.110, *p* = 0.0033]	**Yes** [*F*(1,31) = 5.583, *p* = 0.0246]	No [*F*(1,31) = 0.530, *p* = 0.4721]
11	**Yes** [*F*(1,31) = 8.059, *p* = 0.0079]	No [*F*(1,31) = 1.372, *p* = 0.2504]	No [*F*(1,31) = 1.681, *p* = 0.2044]

The bold “Yes” indicates the significant values.

**FIGURE 4:**
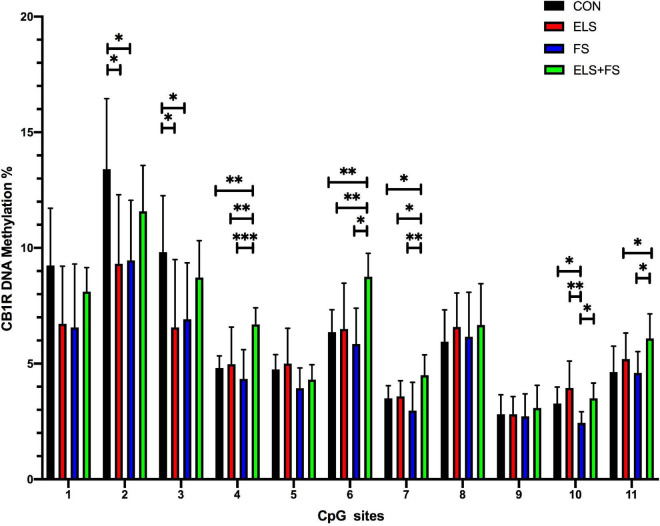
Overview of DNA methylation changes at individual CpG sites within the promoter region of the CB1R gene. SNK *post-hoc* test revealed CpG-site specific significant differences between the experimental groups (*p ≤ 0.05, ^**^p ≤ 0.01, ^***^p ≤ 0.001). All bars depict mean ± SD. CON, control (*n* = 9); ELS, early life stress (*n* = 9); FS, forced swimming (*n* = 8); ELS + FS, early life stress + forced swimming (*n* = 9).

Student Newman Keuls *post-hoc* test indicated several specific significant differences for the analyzed individual CpG sites between the experimental groups (Figure 4). For the ELS group, the results showed significantly lower levels of DNA methylation at CpG sites 2 and 3, when compared to the CON group (*p ≤ 0.05). Similarly, the FS group showed lower DNA methylation, on CpG sites 2, 3, and 10 (*p ≤ 0.05) when compared to the CON group. On CpG site 10, our results show significantly lower methylation levels in the FS group when compared to the ELS group (^**^p ≤ 0.01) and ELS + FS group (*p ≤ 0.05). In addition, the ELS + FS groups showed higher methylation levels than the other groups at CpG sites 4, 6, 7 (*p ≤ 0.05, ^**^p ≤ 0.01, ^***^p ≤ 0.001). At CpG-site 11, ELS + FS showed significantly higher methylation levels, when compared to ELS and FS (*p ≤ 0.05).

#### Fatty acid amide hydrolase (FAAH)

##### FS decreases mean DNA methylation within the FAAH gene promoter

Two-way ANOVA analysis of mean DNA-methylation over the 9 analyzed CpG sites within the promoter region of the FAAH gene revealed a strong effect of FS [*F*(1,31) = 23.52, *p* < 0.0001], but no effect of ELS [*F*(1,31) = 0.01141, *p* = 0.9156], but no effect of ELS [*F*(1,31) = 0.01141, *p* = 0.9156] and no interaction between the two stressors [*F*(1,31) = 1.848, *p* = 0.1838].

**FIGURE 5:**
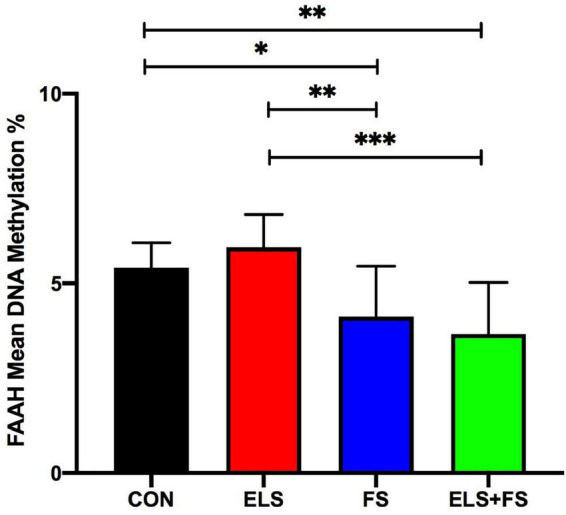
ELS and FS effects on mean DNA methylation levels of 9 CpG sites within the promoter region of FAAH gene. DNA methylation levels are depicted as mean ± SD. Significant results of SNK *post-hoc* test are presented as *p ≤ 0.05, ^**^p ≤ 0.01, and ^***^p ≤ 0.001. CON, control (*n* = 9); ELS, early life stress (*n* = 9); FS, forced swimming (*n* = 8); ELS + FS, early life stress + forced swimming (*n* = 9).

The SNK *post-hoc* test displayed a significant decrease in mean DNA methylation levels in the FS (*p* ≤ 0.05), and ELS + FS (*p* ≤ 0.01) groups, when compared to the CON group (Figure 5) and when compared to the ELS group (FS vs. ELS, *p* ≤ 0.01; ELS + FS vs. ELS, *p* ≤ 0.001). No significant differences were found between the CON and the ELS groups as well as between the FS and the ELS + FS groups.

##### FS induces CpG-site specific decrease in DNA methylation within the FAAH promoter

Two-way ANOVA analysis for each of the analyzed 9 individual CpG sites within the promoter region of the FAAH gene revealed a significant effect for FS but not for ELS and not for ELS + FS (see Table 6 for a summary).

**TABLE 6:** Two-way ANOVA analysis of 9 individual CpG-sites within the promoter region of FAAH gene.

Individual CpG site	Main effect		Interaction
	ELS	FS	ELS + FS
1	No [*F*(1,31) = 0.095, *p* = 0.7601]	**Yes** [*F*(1,31) = 13.930, *p* = 0.0008]	No [*F*(1,31) = 2.091, *p* = 0.1582]
2	No [*F*(1,31) = 0.786, *p* = 0.3821]	**Yes** [*F*(1,31) = 11.720, *p* = 0.0018]	No [*F*(1,31) = 0.0182, *p* = 0.8934]
3	No [*F*(1,31) = 0.566, *p* = 0.4577]	**Yes** [*F*(1,31) = 17.640, *p* = 0.0002]	No [*F*(1,31) = 3.060, *p* = 0.0901]
4	No [*F*(1,31) = 0.001, *p* = 0.9972]	**Yes** [*F*(1,31) = 21.890, *p* < 0.0001]	No [*F*(1,31) = 0.202, *p* = 0.6562]
5	No [*F*(1,31) = 2.121, *p* = 0.1553]	**Yes** [*F*(1,31) = 10.400, *p* = 0.003]	No [*F*(1,31) = 0.029, *p* = 0.8655]
6	No [*F*(1,31) = 0.315, *p* = 0.5785]	**Yes** [*F*(1,31) = 9.425, *p* = 0.0044]	No [*F*(1,31) = 2.021, *p* = 0.1651]
7	No [*F*(1,31) = 0.032, *p* = 0.8577]	**Yes** [*F*(1,31) = 8.090, *p* = 0.0078]	No [*F*(1,31) = 1.073, *p* = 0.3083]
8	No [*F*(1,31) = 0.490, *p* = 0.4892]	**Yes** [*F*(1,31) = 23.640, *p* = < 0.0001]	No [*F*(1,31) = 1.870, *p* = 0.1813]
9	No [*F*(1,31) = 0.003, *p* = 0.9577]	**Yes** [*F*(1,31) = 12.250, *p* = 0.0014]	No [*F*(1,31) = 1.678, *p* = 0.2048]

The bold “Yes” indicates the significant values.

**FIGURE 6:**
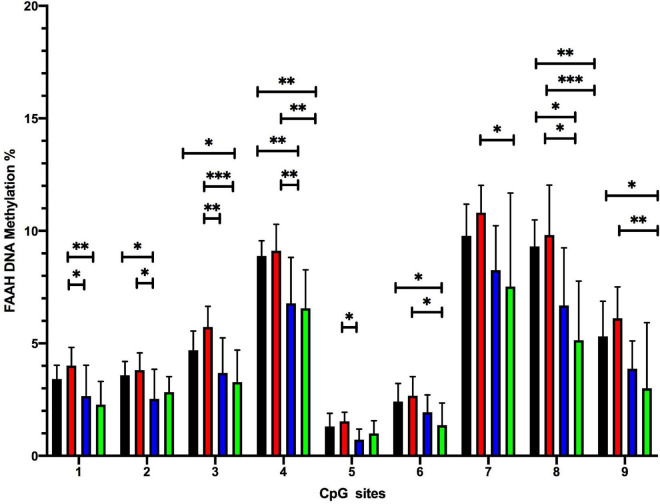
Overview of DNA methylation changes at individual CpG sites within the promoter region of the FAAH gene. SNK *post-hoc* test revealed several CpG-site significant differences between the experimental groups (*p ≤ 0.05, ^**^p ≤ 0.01, and ^***^p ≤ 0.001). CON, control; ELS, early life stress; FS, forced swimming; ELS + FS, early life stress + forced swimming. All bars depict mean ± SD. CON, control (*n* = 9); ELS, early life stress (*n* = 9); FS, forced swimming (*n* = 8); ELS + FS, early life stress + forced swimming (*n* = 9).

Student Newman Keuls *post-hoc* test indicated several CpG-site specific significant differences between the experimental groups (Figure 6). The FS group showed significantly reduced methylation levels at CpG positions 2, 4, and 8 (*p ≤ 0.05, ^**^p ≤ 0.01), when compared to the CON group. The ELS + FS group showed significantly reduced methylation levels at CpG positions 3, 4, 6, 8, and 9 (*p ≤ 0.05, ^**^p ≤ 0.01), when compared to the CON group. When compared to the ELS group, the FS group showed significantly lower methylation levels at CpG positions 1, 2, 3, 4, 5, and 8 (*p ≤ 0.05, ^**^p ≤ 0.01) and ELS + FS showed significantly lower methylation levels at CpG positions 1, 3, 4, 6, 7, 8, and 9 (*p ≤ 0.05, ^**^p ≤ 0.01, ^***^p ≤ 0.001). No significant differences were found between the CON and the ELS group as well as between the FS and the ELS + FS groups.

## Discussion

Exposure to adverse experiences prenatally, in early life and adolescence critically influence the functional maturation of the nervous system and thus represent an important programming factor for the development of mental health and disease in later life periods (Heim and Nemeroff, 2001; Bock et al., 2014, 2015; Babenko et al., 2015; Braun et al., 2020). However, the detailed mechanisms that moderate dysfunctional versus protective and resilient adaptations are still unclear. A critical factor that determines the magnitude and direction (detrimental or adaptive) of long-term behavioral and neuronal/synaptic consequences of stress is age, i.e., the maturity of the brain. Thus, one aim of the present study was to investigate the effect of stress exposure during two developmental time windows, which are characterized by highly active synaptic development and reorganization. We specifically addressed the question in which way early postnatal stress (ELS) and swim stress during adolescence (FS) as single stressors may differentially affect the development of the endocannabinoid system in the mPFC, with focus on the expression of CB1R and FAAH genes. We show here in adult female rats that gene expression of CB1R was upregulated by exposure to ELS as well as by exposure to FS, whereas FAAH gene expression was upregulated only by FS. Another aim was to investigate if ELS exposure, i.e., stress during a very immature stage of cortical development, might induce a long-term “reprogramming” of gene expression and thereby either amplify or ameliorate the cellular response toward FS. We observed that ELS indeed suppressed the FS-induced upregulation of both genes. Finally, we addressed the hypothesis that these stress-induced changes of CB1R and FAAH gene expression are mediated by epigenetic modifications, specifically through DNA methylation. In support of this hypothesis we found in adult animals that elevated gene expression was paralleled by decreased DNA-methylation in the promoter region of the respective gene.

### The endocannabinoid system is affected by early life stress and adolescent stress

We observed that exposure to a single stressor, either during a very early postnatal time window (ELS) or during a later developmental period, adolescence (FS), induced changes in the endocannabinoid system in the mPFC, which are maintained until adulthood. With respect to the CB1R gene expression, we found that both stressors, ELS and FS, induced a long-term upregulation when compared to unstressed controls. This observation is in line with studies, which reported enhanced CB1R gene expression and elevated receptor density in the PFC of rats after chronic mild stress adolescence (Hillard et al., 2006; Bortolato et al., 2007; Hill et al., 2008; McLaughlin et al., 2014). It has also been shown that repeated restraint stress during adolescence results in increased CB1R density in the PFC of adult male rats (Lee and Hill, 2013). In this study, a CB1R upregulation after adolescence stress was also found in the amygdala but not in the hippocampus, indicating potential region-specific effects (Lee and Hill, 2013).

The question arises if the detected upregulation of CB1R expression reflects a dysfunctional or rather a positive or protective adaptation. Deficiency of CB1 receptor signaling has been claimed to be associated with anhedonia and anxiety (Hillard, 2014) since CB1Rs are expressed on glutamatergic, GABAergic serotonergic and noradrenergic axon terminals in different brain areas and thus are involved in the regulation of these neurotransmitters that are critically implicated in the control of stress and socio-emotional behavior. Pathological changes of CB1R in the mPFC were reported in post-mortem brains of suicide victims previously diagnosed with major depression, which showed increased CB1R density and a higher binding affinity (Hungund et al., 2004; McLaughlin et al., 2014). Thus, it is tempting to speculate that a ELS-induced change of CB1R expression, as found in our study in the mPFC, may result in an imbalance of excitation and inhibition and impaired brain function in this brain area and as a consequence in disturbed behavioral profiles. This is supported by our findings that the ELS-exposed animals show symptoms of anxiety- and depressive-like behavior and slight disturbances of social interaction, which is in line with previous findings applying this particular ELS paradigm (Alteba et al., 2016, 2020, 2021; Portugalov et al., 2022). On the other hand, a potential neuroprotective role of CB1R signaling was proposed, since stress-induced CB1R activation, specifically on glutamatergic terminals, may protect from excitotoxicity and neuronal cell death (Zoppi et al., 2011).

There is evidence that the outcome of stress exposures with respect to the endocannabinoid system appears to be region-specific and dependent on the developmental timepoint when the respective stressor was experienced. Chronic adult stress experience induced different effects than an adolescent stressor, i.e., adult stress exposure induced an increase of CB1R density only in the PFC, whereas adolescent stress exposure resulted in a downregulation of this receptor in the hippocampus (Lee and Hill, 2013). The concept that the effects of stress on the brain are critically dependent on the developmental time window during stress exposure (Lupien et al., 2009; Bock et al., 2015) is further supported by our observation that FAAH expression was upregulated only in response to FS exposure during adolescence, but not after ELS.

Our study also revealed a positive correlation of CB1R and FAAH expression, i.e., higher CB1R gene expression was accompanied by higher FAAH gene expression. A similar positive correlation of CB1R and FAAH mRNA expression was also found in a post-mortem analysis in the PFC of healthy individuals (Muguruza et al., 2019). We have to consider that the positive correlation between the two genes found in our study describes the correlation over all individuals of all individual groups. Here the correlation is mainly due to the increased expression levels of eCB1 in the ELS and FS group and the increased levels of FAAH in the FS group when compared to the other two groups. However, focusing on the individual experimental groups we find a trend for a positive correlation only in the control and FS groups. In animals exposed to ELS this positive correlation between the genes was completely absent, potentially reflecting an altered balance in the regulation of the endocannabinoid system, which may contribute to proposed programming effects of ELS.

### ELS suppresses FS-induced upregulation of CB1R and FAAH gene expression

Guided by the “two-(or multiple) hit” concept (Daskalakis et al., 2013; Gruber et al., 2015; Lesse et al., 2017; Kocamaz et al., 2022) we hypothesized that ELS might influence the observed upregulation of CB1R and FAAH gene expression in response to FS exposure during adolescence. Our and other studies revealed that vulnerable as well as resilient adaptations may occur depending on the time point of stress exposure, and that the presumed “(re)programming” effects of the first stressor may either amplify or ameliorate/suppress the cellular and molecular responses toward subsequent stressors (Nederhof and Schmidt, 2012; Daskalakis et al., 2013; McElroy and Hevey, 2014). In the present study we found that gene expression levels in the ELS + FS group remained similar to those of the control group and was significantly lower compared to gene expression in response to a single FS exposure. This observation supports the hypothesis that exposure to ELS induces a “(re)programming” effect and thereby suppressed the FS-induced changes in gene expression. In line with this interpretation our study also reveals on the behavioral level that FS-induced enhanced anxiety-like behavior in the OF-test is reversed by pre-exposure to ELS. Additionally, the increased number of affected animals in the FS group detected by our behavioral profiling approach was reversed back nearly to control levels in the ELS + FS group. The interpretation of a “(re)programming” effect of ELS is further supported by findings of our previous studies in male mice on hippocampal NPY-Y2 receptor and oxytocin receptor (OxtR) gene expression. Similar to the “buffering” effect of ELS observed in the present study we found increased NPY-Y2 gene expression after a single adult swim stress exposure, whereas animals exposed to both stressors (ELS + AS) displayed expression levels similar to those in unstressed controls (Kocamaz et al., 2022). For OxtR gene expression we found evidence for an “amplifying” effect of ELS: while ELS or AS alone had no effect on gene expression, the consecutive exposure to both ELS and AS resulted in increased gene expression in the hippocampal formation (Lesse et al., 2017). “(re)programming” effects of a first stressor were also reported in a recent study in rats, which investigated the influence of adult chronic restraint stress on modifications induced by the exposure to a second stressor. This study found that chronic restraint stress influenced the release of corticosterone induced by a second acute restraint episode, and inhibited the ability of the second stressor to activate brain-derived neurotrophic factor (BDNF) in the PFC (Brivio et al., 2020). Another study reported an interaction between social defeat stress during early adolescence and prolonged stress in adulthood: adolescent stress normalized protein levels of BDNF in the hippocampus in response to adult stress exposure (Mancini et al., 2021).

The detailed mechanisms underlying the complex interaction of the multiple stressors experienced throughout life are far from understood. The question remains in which way the exposure to one stressor, (re)programs” the response to consecutive stress experiences later in life. An additional view on the nature of the long-term changes of ELS-induced upregulation of gene expression may be derived from evidence that endocannabinoids may interfere with the development and maturation of excitatory and inhibitory synaptic connectivities in the postnatal brain (Maccarrone et al., 2014). Thus, the detected ELS-induced lasting upregulation of CB1R and FAAH gene expression may result in a change of the excitation/inhibition balance in the mPFC and thereby perhaps influences its capacity to cope with stress later in life. During ELS exposure the majority of synapses in the mPFC are still immature and the growth and establishment of synaptic circuits is still ongoing. Endocannabinoids orchestrate synaptic growth and signaling as well as directional axon growth by binding to their target receptors, including CB1R. Thereby, the availability of endocannabinoids is determined by biosynthesis enzymes as well as by degrading enzymes, including FAAH and others (Maccarrone et al., 2014), whose expression/activity may confer an additional modulatory influence during synaptic development and maturation. Hence, it may be speculated that the ELS-induced changes in the endocannabinoid system may promote the establishment of functional synaptic circuits in the mPFC, and thereby influence stress responses and emotional behavior later in life.

### Early life stress and adolescent stress change DNA methylation pattern on the promoter region of endocannabinoid system genes

There is increasing evidence that the effects of ELS on brain and behavioral functions are mediated *via* epigenetic mechanisms that result in altered gene expression profiles (Uchida et al., 2010; Xie et al., 2013; Alyamani and Murgatroyd, 2018; Li et al., 2020).

Among the different epigenetic modifications such as histone modifications, microRNAs and DNA methylation, the latter is one of the best-described mechanisms with respect to epigenetic changes induced by early adverse experiences (Park et al., 2014; Sasagawa et al., 2017; Zhang et al., 2019; Kocamaz et al., 2022). This epigenetic modification represents a crucial regulator for the fine-tuning of gene expression and the classical view is that enhanced DNA-methylation (hypermethylation) leads to decreased or even silenced gene expression (Bird, 2002; Greenberg and Bourc’his, 2019). DNA-methylation can regulate and mediate the effects of temporary exposure to early adverse situations into stable alterations in gene expression after the initial exposure has ended (Nuyt and Szyf, 2007).

In the present study, we focused on DNA methylation at specific CpG sites in the promoter regions of the CB1R and FAAH genes. Coinciding with CB1R gene expression results, enhanced expression in the ELS and FS groups, mean DNA methylation across the analyzed CpG-sites was decreased in these two experimental groups, when compared to the control group. Further evidence that the detected CB1R gene expression changes are, at least partly mediated by altered DNA-methylation is gained from our results that show a clear negative correlation of gene expression with mean DNA-methylation across all experimental groups. However, our additional CpG-site specific analysis revealed that not all analyzed CpG sites are affected in the same manner, but the stress-induced changes were restricted to some circumscribed sites. Also, conceding with FAAH gene expression results, increased expression in the FS group, we found decreased mean DNA-methylation across the analyzed CpG-sites in the FAAH promoter. The additional CpG-site specific analysis revealed that all analyzed sites were affected by FS exposure. In sum, our data indicate that ELS as well as FS at adolescence induced specific, stable changes of DNA-methylation at the promoter regions of CB1R and FAAH that last into adulthood and may contribute to the detected changes of gene expression of these two major factors of the endocannabinoid system. Epigenetically mediated changes of factors within the endocannabinoid system have also been described in other studies, including the effects of stress or stress-associated diseases. For example, chronic and unpredictable maternal separation induced changes of DNA methylation in a number of genes, including the CB1R gene, in the germ cells of male mice. Since the same methylation patterns were found in the cortex of F2 generation offspring, this indicates a possible transgenerational transmission of the stress-induced epigenetic changes (Franklin et al., 2010). However, it has to be considered that the interfering effects of stress on signaling pathways in the brain are often mediated by an interaction of DNA-methylation with other epigenetic modifications such as histone modifications (Bagot et al., 2012; Greenberg and Bourc’his, 2019). With regard to the endocannabinoid system, it has been shown that unpredictable, mild stress in male mice resulted in decreased H3K9 acetylation at the FAAH promoter in the hypothalamus, associated with reduced gene expression (Pucci et al., 2019). Another study demonstrated that chronic unpredictable stress in male mice decreased CB1R gene expression in the cingulate cortex, paralleled by reduced H3K9 acetylation at the CB1R gene (Lomazzo et al., 2017).

### ELS pre-exposure suppressed FS-induced changes of DNA-methylation at the CB1R and FAAH gene

Matching our gene expression results within the context of the two (multiple-)hit model, our study also reveals that the exposure to ELS reversed the reduced mean DNA-methylation levels of both analyzed genes back to control levels. However, our CpG-site specific analysis revealed, particularly for the CB1R gene, that DNA-methylation levels were not identical when comparing the CON and the ELS + FS group, since some of the analyzed CpG-sites were significantly different between the two groups. Thus, it is tempting to speculate that these subtle, well-defined CpG-site specific changes in DNA-methylation may reflect a possible mechanism underlying an ELS-induced long-term “programming” toward later stress exposures and in the present study this may protect the ELS pre-exposed animals against the gene expression changes in response to FS at adolescence. This hypothesis is in line with a model for epigenetic programming stating that experience triggers specific epigenetic alterations that anticipate responses to future signals (Szyf, 2019). Such epigenetic changes, as for example demethylation of specific CpG-sites triggered by the action of stress-hormones, are presumably insufficient for an activation of the affected genes, however, they are potentially predictive (programming) for the response and alterations of these genes to consecutive stressful events at later life periods.

## Conclusion

Our study reveals that stress experienced at two critical developmental time windows, the early postnatal period and adolescence, affects the expression of main elements of the endocannabinoid system, CB1R and FAAH, in the mPFC of adult female rats. These expression changes are paralleled and presumably at least partly mediated by changes of DNA-methylation levels at the promoter region of these genes. Moreover, our findings indicate that ELS can act as a (re-)programming factor toward the responses to subsequent stress experiences at adolescence. This (re-)programming effect may be reflected in subtle alterations of DNA-methylation levels at circumscribed CpG-sites. However, future studies are required to investigate the underlying mechanisms of the described consequences in response to single and multiple stress exposures at critical developmental time windows in more detail. Particularly, it has to be assessed if the observed gene expression changes in the endocannabinoid system may contribute to the development of pathological or resilient behavioral phenotypes later in life.

## Data availability statement

The original contributions presented in this study are included in this article/supplementary material, further inquiries can be directed to the corresponding author.

## Ethics statement

The experiments were approved by the University of Haifa Ethics and Animal Care Committee and appropriate measures were taken to minimize pain and discomfort (approval numbers: 741/20 and 765/21).

## Author contributions

AD conducted the gene expression experiments, assessment of DNA-methylation levels, the according statistical analysis, and wrote significant parts of the manuscript including preparation of figures. MD and AP conducted the animal experiments including stress exposure and collected brain samples for analysis. IA and MM were involved in planning of the project, supervised the animal experiments (stress exposure and collection of brain samples), corrected and edited significant parts of the manuscript, and provided partial funding (DFG, BR 1692/11-1). KB supervised the project, planned the experimental design and wrote and edited significant parts of the manuscript, and provided funding (DFG, BR 1692/11-1). JB supervised and planned the project and experimental design, was involved in the statistical analysis, wrote and edited large parts of the manuscript, and provided funding (DFG, BO 1674/7-1). All authors contributed to the article and approved the submitted version.
